# Evaluation of freely available artificial intelligence (AI) tools for diagnostic support in a complex clinical and laboratory presentations: a pilot study

**DOI:** 10.1515/almed-2025-0157

**Published:** 2025-10-29

**Authors:** Laura Pighi, Davide Negrini, Giuseppe Lippi

**Affiliations:** Section of Clinical Biochemistry, University of Verona, Verona, Italy

**Keywords:** artificial intelligence, ChatGPT, Perplexity, GEMINI

To the Editor,

Artificial intelligence (AI) is increasingly influencing daily life, scientific research, and medical practice. As AI tools continue to evolve and become freely accessible, their use in clinical settings is rapidly expanding [1]. Recent evidence highlights the potential of AI to support diagnostic decision-making. For example, a recent clinical trial in which participants were randomized to either access ChatGPT Plus (GPT-4) alongside conventional diagnostic resources or rely only on conventional resources for reviewing six clinical vignettes within 60 min [2], revealed that the AI tool by itself outperformed both physician groups, highlighting the potential implications of integrating such digital tools into routine medical practice.

To this end, we conducted a pilot study to explore the diagnostic performance of three freely available AI tools in interpreting a complex clinical scenario: an older patient with mandibular pain lasting for around 1 h, which may represent either an initial atypical presentation of non-ST-elevation myocardial infarction (NSTEMI) or dental-origin mandibular pain. Atypical pain presentations of NSTEMI in older adults, such as mandibular discomfort, are well recognized and can complicate the final diagnosis, especially when the interval between symptom onset and cardiac troponin testing is relatively short [3].

The following AI tools were evaluated: Gemini AI 2.5, ChatGPT 5.0, and Perplexity 2.250911.0. Each AI was queried with the same case information:

– Age: 75 years
– Sex: male
– Body mass index: 32 kg/m^2^
– Symptoms: Mandibular pain (intensity 7/10) radiating to the neck, onset 1 h prior to hospital admission
– Body temperature: 36.2 °C
– Heart rate: 94 bpm
– Respiratoryrate: 37/min
– ECG: normal
– Hemoglobin: 129 g/L (reference range >130 g/L)
– Plasma glucose: 4.2 mmol/L (reference range 3.9–5.5 mmol/L)
– Arterial lactate: 2.1 mmol/L (0.5–2.2 mmol/L)
– Cardiac troponin T: 21 ng/L (reference range <22 ng/L)

The AI tools were asked to suggest the most likely diagnoses and additional diagnostic tests.

The results of our investigation are summarized in Table 1. Briefly, all three AI tools correctly identified acute coronary syndrome (ACS) or unstable angina as the most likely diagnosis. All three AI tools generated a broader differential diagnosis that included both cardiac and non-cardiac causes, including acute aortic syndrome (dissection) and pulmonary embolism, as well as other non-cardiac conditions such as temporomandibular joint disorders or dysfunction, dental pathologies, neuralgia, infections, and musculoskeletal pain. Regarding diagnostic evaluations, ChatGPT recommended serial high-sensitivity troponin measurements, repeat ECGs, chest X-ray, and echocardiography. Perplexity suggested serial high-sensitivity troponin testing, echocardiography, coronary CT angiography or invasive coronary angiography, and chest X-ray. GEMINI proposed a narrower set of predominantly cardiac-focused assessments, including serial troponin testing, coronary angiography, echocardiography, stress testing, cardiac CT angiography, and even a lipid panel.

**Table 1:** Output from three freely available artificial intelligence (AI) tools in response to a complex case of atypical presentation (i.e., mandibular pain) potentially associated with non-ST elevation myocardial infarction (NSTEMI) in an older adult.

Output	ChatGPT	Perplexity	GEMINI
Most likely diagnosis	Early or evolving acute coronary syndrome (ACS), possibly non-ST-elevation myocardial infarction (NSTEMI)	Acute coronary syndrome (ACS) presenting atypically with orofacial pain	Unstable angina, a type of acute coronary syndrome (ACS)
Other potential diagnoses	– Angina pectoris (unstable angina) – Acute aortic syndrome (dissection) – Pulmonary embolism – Dental or temporomandibular joint pathology – Musculoskeletal pain	– Temporomandibular joint disorders – Neuralgia – Infections – Musculoskeletal causes	– Temporomandibular joint disfunction – Dental abscess or infection – Aortic dissection
Additional tests	– Serial high-sensitivity troponin – Repeat ECGs – Chest X-ray – Echocardiography	– Serial high-sensitivity cardiac troponin testing – Echocardiography – Coronary CT angiography or invasive coronary angiography – Chest X-ray	– Serial cardiac troponin testing – Coronary angiography – Echocardiography – Stress test – Lipid panel – Cardiac computed tomography (CT) angiography

This pilot study demonstrates that freely available AI tools, such as Gemini AI 2.5, ChatGPT 5.0, and Perplexity 2.250911.0, can support diagnostic reasoning in complex cases, including atypical mandibular pain, which may potentially reflect NSTEMI in older adults. All three AI tools consistently identified myocardial ischemia as the most likely diagnosis, prioritizing life-threatening conditions despite atypical symptoms and a negative result of cardiac troponin T (attributed to early presentation). The three AI tools also considered a broad range of cardiac and non-cardiac causes, including aortic syndrome, pulmonary embolism, temporomandibular disorders, dental infections, neuralgia, and musculoskeletal pain. Recommendations for further testing were in line with current guidelines (i.e., repeated cardiac troponin testing) [4], and varied as ChatGPT balanced cardiac and supportive imaging, Perplexity emphasized advanced coronary imaging, while Gemini focused on more cardiology-specific tests. These differences likely reflect variations in model training or algorithms.

In conclusion, our findings show that AI tools have the potential to identify critical conditions like ACS and guide diagnostic strategy. Although promising for aiding clinicians, especially those less experienced or time-constrained, AI outputs are intended to supplement and not replace clinical judgment since clinical expertise remains crucial for guaranteeing safe and effective patient care.

## References

[1] Lippi G, Plebani M (2025). Lights and shadows of artificial intelligence in laboratory medicine. Adv Lab Med.

[2] Goh E, Gallo R, Hom J, Strong E, Weng Y, Kerman H (2024). Large language model influence on diagnostic reasoning: a randomized clinical trial. JAMA Netw Open.

[3] Alexander KP, Newby LK, Cannon CP, Armstrong PW, Gibler WB, Rich MW (2007). Acute coronary care in the elderly, part I: non-ST-segment-elevation acute coronary syndromes: a scientific statement for healthcare professionals from the American heart association council on clinical cardiology: in collaboration with the society of geriatric cardiology. Circulation.

[4] Byrne RA, Rossello X, Coughlan JJ, Barbato E, Berry C, Chieffo A (2023). 2023 ESC guidelines for the management of acute coronary syndromes. Eur Heart J.

